# Development and launch of the first obstetrics and gynaecology master of medicine residency training programme in Botswana

**DOI:** 10.1186/s12909-020-02446-1

**Published:** 2021-01-06

**Authors:** R. Luckett, M. Nassali, T. Melese, B. Moreri-Ntshabele, T. Moloi, G. J. Hofmeyr, K. Chobanga, J. Masunge, J. Makhema, M. Pollard, H. A. Ricciotti, D. Ramogola-Masire, L. Bazzett-Matabele

**Affiliations:** 1 Department of Obstetrics and Gynaecology, Faculty of Medicine, University of Botswana, Gaborone, Botswana; 2 Department of Obstetrics and Gynaecology, Princess Marina Hospital, Gaborone, Botswana; 3 The Botswana Harvard AIDS Institute Partnership, Gaborone, Botswana; 4 Department of Obstetrics and Gynecology, Beth Israel Deaconess Medical Center, Boston, USA; 5 Harvard Medical School, Boston, USA; 6 Faculty of Medicine, University of Botswana, Gaborone, Botswana

## Abstract

**Background:**

Sub-Saharan Africa (SSA) faces a severe shortage of Obstetrician Gynaecologists (OBGYNs). While the Lancet Commission for Global Surgery recommends 20 OBGYNs per 100,000 population, Botswana has only 40 OBGYNs for a population of 2.3 million. We describe the development of the first OBGYN Master of Medicine (MMed) training programme in Botswana to address this human resource shortage.

**Methods:**

We developed a 4-year OBGYN MMed programme at the University of Botswana (UB) using the Kern’s approach. In-line with UB MMed standards, the programme includes clinical apprenticeship training complemented by didactic and research requirements. We benchmarked curriculum content, learning outcomes, competencies, assessment strategies and research requirements with regional and international programmes. We engaged relevant local stakeholders and developed international collaborations to support in-country subspecialty training.

**Results:**

The OBGYN MMed curriculum was completed and approved by all relevant UB bodies within ten months during which time additional staff were recruited and programme financing was assured. The programme was advertised immediately; 26 candidates applied for four positions, and all selected candidates accepted. The programme was launched in January 2020 with government salary support of all residents. The clinical rotations and curricular development have been rolled out successfully. The first round of continuous assessment of residents was performed and internal programme evaluation was conducted. The national accreditation process was initiated.

**Conclusion:**

Training OBGYNs in-country has many benefits to health systems in SSA. Curricula can be adjusted to local resource context yet achieve international standards through thoughtful design and purposeful collaborations.

## Background

The global burden of maternal morbidity and mortality falls disproportionately on low- and middle-income countries (LMICs), and women bear the greatest burden of limited access to comprehensive health services [1, 2]. In particular, surgical access for obstetric emergencies, reproductive health problems, and gynaecologic malignancies in LMICs falls far short of international targets [3]. Outmigration of health workers from LMICs contributes to the global disparity and counteracts efforts to improve health systems [4, 5].

One contributing factor to disproportionate access in sub-Saharan African countries is limited in-country specialty training opportunities [6, 7]. The *Lancet Commission for Global Surgery* recommends a ratio of at least 20 OBGYNs per 100,000 population [8]. In 2011, there were an estimated 250 OBGYN residency training positions for the 7800 medical students graduating from African medical schools serving nearly 1 billion people [9]. In-country postgraduate training is known to improve retention of doctors [10]. When post-specialization employment opportunities exist, specialist retention enhances clinical care, improves local leadership, and specifically for OBGYNs, contributes to maternal mortality reduction [11, 12].

Like many LMICs, Botswana faces physician shortages, with 37 doctors per 100,000 population [13]. The maternal mortality ratio in 2017 was 143 per 100,000, more than twice the Sustainable Development Goal target for 2030 [14]. In the fifteen years preceding the establishment of Botswana’s first medical school, approximately 1000 Batswana were sponsored for undergraduate medical education abroad, but only 10% returned [15]. Data are not available on Batswana doctors sent abroad for OBGYN specialty training, but currently for the population of 2.3 million people, only 40 OBGYNs are practicing clinically in-country. Of these 40 OBGYNs, only 12 practice in the public sector where a majority of the population seeks care (personal communication between RL and OBGYN practitioners in the country) [16]. Most OBGYNs are foreign nationals; only 6 Batswana OBGYNs practice in the country, of whom only 2 practice in the public sector.

Here, we describe the development and launch of the first OBGYN Master of Medicine (MMed) residency training programme in Botswana. The aim of the programme is to establish high quality OBGYN training in-country to address the human resource shortage within the country. Beyond producing well-trained specialists, the programme aims to develop leaders in the field to streamline standard-of-care practice in the country and advance the health of women in Botswana. The aim of this manuscript is to detail the approach, processes and contextual considerations as a guide for other institutions who seek to build and strengthen OBGYN specialty training across Africa.

## Methods

### Setting

The University of Botswana (UB) established the only medical school in the country in 2009 and is the only institution offering post-graduate medical training. UB has a clear process for programme approval and pathway to national accreditation, with existing MMed residency training programmes in Internal Medicine, Paediatrics, Family Medicine, Emergency Medicine, Pathology, Anaesthesia and Public Health. Except for Family Medicine and Public Health, all MMed training programmes are “sandwich” programmes, with partial training in Botswana and more specialized tertiary and quaternary training in South Africa. OBGYN aimed to create a fully in-country MMed programme that meets international curricular standards, anticipates national and international accreditation, and enables further sub-specialization.

We approached our OBGYN MMed training programme development using the Kern’s 6-step approach namely: 1) general needs assessment, 2) targeted needs assessment, 3) delineating learning outcomes, 4) designing educational strategies, curricular structure and content, 5) implementing the curriculum and 6) evaluating the curriculum [17].

### Needs assessment

The Faculty of Medicine conducted a general needs assessment for medical training in the country prior to establishing the medical school. They reviewed key government documents and policies, Botswana Health Professions Council records, hospital staffing levels, availability of citizen doctors and specialty service availability. Their findings demonstrated a lack of available specialty services and a public desire for access to Batswana doctors.

The department of OBGYN conducted a targeted needs assessment specific to OBGYN and women’s health in the country. The Botswana Ministry of Health and Wellness’ (MOHW) Integrated Health Service Delivery Strategic Plan for 2010–2020 delineated their priorities for the health services sector, including achieving optimal comprehensive sexual and reproductive health for women and ensuring access to high quality antenatal and perinatal care [18]. Principal challenges preventing achievement of the strategic objectives included excessive shortage of skilled human resources, poor quality of care, and lack of quality management and regulation in both the public and private sector. Particularly for OBGYN, the few specialists working in the public sector only staff 4 of the 31 hospitals where the general public can seek free care. The waiting time to see a specialist at the tertiary referral hospital in the Southern Region is at least eight months, and surgical waiting times after evaluation is another eight to twelve months. There is limited access to sub-specialty gynaecologic care. There is one Gynaecologic Oncologist who provides care in the public sector and one Reproductive Endocrinologist who offers services in the private sector. There are no maternal-fetal medicine, urogynaecology, nor minimally invasive gynaecologic surgical specialists in the country, and no pipeline of trained OBGYNs to send to these training programmes.

In addition to the lack of specialists, there is no regulation of the quality of services provided, nor national standard OBGYN practice guidelines.

The OBGYN MMed programme was developed in consultation with relevant stakeholders. Involvement of the MOHW, Botswana Health Professions Council, and the Botswana Qualifications Authority ensured that the programme complies with the national vision for comprehensive sexual and reproductive health services and meets national accreditation standards. Input from OBGYN specialists with diverse training backgrounds from both public and private sectors in Botswana ensured that our curriculum aligned with practice expectations. Departments associated with academic programme development at UB and departments concurrently developing MMed programmes provided essential guidance on the process.

Finally, we assessed the demand for specialty training in OBGYN by involving potential future trainees in consultative meetings with stakeholders. In addition to the five cohorts of graduated medical students from UB, there are Batswana and non-Batswana graduates of foreign medical schools who are working in the country while awaiting opportunities to specialise. In-country training is an attractive option to many, particularly those with families and other personal commitments in Botswana.

### Learning outcomes

Six essential learning outcomes were delineated in-line with international graduate medical education standards, alongside evaluation criteria that guide resident assessment of outcome attainment [19]. The learning outcomes were aligned and mapped to the Botswana Health Professions Council and Botswana Qualifications authority guidelines for accreditation of a programme. A formal comparison of the UB curriculum to regional and international training programmes in regards to learning outcomes, curriculum domains covered, credit weighting, assessment strategy, qualification requirements and employment pathways was produced. Additionally, specific minimum standards for procedure numbers and competence were delineated for general OBGYN skills and benchmarked against international programmes.

### Educational strategy, curriculum structure and content

All clinical post-graduate medical training programmes at UB are designed as MMed programmes, and include didactics, clinical apprenticeship training, and research requirements. The OBGYN programme is structured as a 4-year integrated and spiralling clinical apprenticeship and capitalizes on the infrastructure of existing MMed programmes at UB, particularly for courses beyond the OBGYN-specific course content (Table 1). The spiralling nature of the curriculum allows residents initial exposures to curricular content through clinical work and didactics, and then repeated exposure allowing deeper understanding during their training. The spiralling approach is purposefully applied to deliver increasingly complex content, at various learning events across multiple levels of the training. Residents are expected to mature in competence and confidence as they progress through the programme. Advanced clinical knowledge and skill is accompanied by graded clinical responsibility fostered through a multi-year student and resident team structure, led by senior residents.

**Table 1:** The Obstetrics and Gynaecology Master in Medicine course sequence

Course title	Total Hours	UB^**a**^ credits
**Semester 1**		
Communication, ethics and professionalism	140	2
Introduction to Obstetrics and Gynaecology I	1300	22
**Semester 2**		
Introduction to clinical research	140	2
Introduction to medical literature	140	2
Introduction to Obstetrics and Gynaecology II	1160	20
**Semester 3**		
Principles and techniques of medical education	140	2
Dissertation I	140	2
Intermediate Obstetrics and Gynaecology I	1160	20
**Semester 4**		
Public health principles and international health	140	2
Dissertation 2	140	2
Intermediate Obstetrics and Gynaecology II	1160	20
**Semester 5**		
Introduction to healthcare management	140	2
Dissertation 3	140	2
Advanced Obstetrics and Gynaecology I	1160	20
**Semester 6**		
Dissertation 4	140	2
Presentation and Defense of Dissertation	140	2
Advanced Obstetrics and Gynaecology II	1160	20
**Semester 7**		
Presentation and Defense of Dissertation II	140	2
Advanced Obstetrics and Gynaecology III	**1300**	22
**Semester 8**		
Advanced Obstetrics and Gynaecology IV	1440	24

^a^*UB* University of Botswana

An example of the spiralling curriculum is how we planned to teach the topic of labour. In the course *Introduction to OBGYN* in year one, the resident will master knowledge and skills in the mechanism and physiology of labour, as well as the management of labour. In their clinical rotations they will participate in care on the labour ward. In the course *Intermediate OBGYN* in year two, they are expected to understand management of normal and abnormal labour while additionally mastering operative vaginal delivery and labour complicated by malpresentation. They will have increased responsibility in their clinical rotations on labour ward and be expected to perform supervised procedures, operative vaginal delivery and external cephalic version. Finally, in *Advanced OBGYN* in years three and four, the resident will be expected to understand and independently manage pregnancy complications such as preterm prelabour rupture of members, high-risk medical conditions in pregnancy and coordinate interdisciplinary teams managing high-risk pregnancies. In their clinical rotations, they will lead and teach junior residents and medical students in antepartum care and on labour ward.

OBGYN curriculum development required review of content and competencies from regional and international OBGYN training programmes. In addition to review of formal curricula, a regional benchmarking visit to the University of Cape Town provided the opportunity for discussion and clarification of essential and advanced content available at a rigorous programme in the region. Considering regional and international curriculum standards, we created our unique curriculum in accordance with UB templates and requirements for proposal of a new academic programme. After refinement of overall curriculum, the content was allocated to semester courses within the UB MMed framework to guide educational programming. Clinical rotations were purposefully designed to provide exposure to course content, provide strong core and subspecialty OBGYN training, as well as exposure to other essential fields (such as surgery and radiology).

Learning outcomes and curriculum content were mapped to educational events in specific courses to ensure the delivery of courses would facilitate attainment of the specified learning outcomes and requisite curricular content to become competent independent practitioners. The OBGYN specific courses in the MMed curriculum were designed as synchronous apprenticeship-based learning. Residents are in the clinical setting on a near daily basis. Clinical learning events include daily specialist ward rounds, precepted outpatient clinics, and supervised theatre cases. Residents have the opportunity to work one-on-one with specialists, as well as together in a team of learners of all levels of the medical training hierarchy. The graduate medical education courses required for all MMed programmes at UB are designed as week-long intensive courses and residents are excused from clinical duties during these courses.

UB Faculty of Medicine uses asynchronous learner-centred, problem-based curriculum, and this framework guided the design of didactics [15]. Learning is largely self-directed*,* complemented by two hours per week of synchronous faculty-facilitated discussions or resident presentations. Additional synchronous learning events include monthly journal clubs, patient management sessions, research supervision sessions, and maternal and perinatal morbidity and mortality conferences. Among these sessions, opportunities for interdepartmental engagement with Surgery, Emergency Medicine, Paediatrics and Anaesthesia allow relevant interdisciplinary topics to be explored.

### Programme regulations, trainee assessment and programme evaluation

The programme operates under the general regulations of the UB School of Graduate Studies and the Faculty of Medicine. Continuous and summative assessments enable formal resident feedback. Biannual continuous assessment uses the milestone framework, in addition to logbook evaluation [20]. Summative assessment is designed as an internally and externally moderated annual examination. Engagement in research and submission of an original thesis is a qualification requirement for all UB MMed candidates. Opportunities for remediation and requirements for progression are clearly elaborated.

Annual programme and faculty evaluations were designed to be administered through anonymous resident surveys. Additionally, residents are provided the opportunity to give confidential direct feedback at their biannual continuous assessment reviews with programme leadership. This feedback and evaluation allow the opportunity for the programme to respond dynamically to meet the needs of its trainees and improve the quality of delivery of its content.

### Resources and infrastructure

Increased OBGYN department staffing to serve as clinician educators was identified as essential to the successful rollout of the programme. The department had 4 OBGYN generalists at initiation of design of the programme, from Ethiopia, Tanzania, Uganda, and the United States of America. Purposeful faculty recruitment to strengthen clinical, educational and research activities in the department was undertaken, with the aim of attaining a 2:1 trainee to teacher ratio.

Clinical sites were considered based on volume and variety of cases to ensure adequate exposure to the curricular content. The primary training site was determined to be the largest referral hospital in the country, which has 570 beds, an adequate variety of cases, adequate theatre volume, essential specialty clinics, and high-volume inpatient wards. Other specialities including surgery, radiology, anaesthesia and intensive care which support optimal OBGYN training are also available on-site. Full-time UB OBGYN faculty supervise and teach residents. Additional training sites would be considered in the future, given adequate supervision, volume, and acuity. UB has a well-resourced library with comprehensive resources, including books, journals, electronic databases, and mobile applications.

UB had allocated funding for increased senior-level staffing. Additional costs were anticipated for visiting scholars to supplement subspecialty training. Funding for resident salaries, tuition and accommodation was sought from the MOHW.

## Results

The learning outcomes, corresponding curriculum and assessment strategy for the OBGYN MMed programme was intensively prepared in three months and finalized by the OBGYN Department in March 2019 (Table 2). The Faculty of Medicine Executive Committee approved the programme in April 2019, alongside feedback from the resource planning committee to assure support through existing funding streams. The UB Senate and Council approved the programme in August and October 2019, respectively. Concurrent with the programme approval process, key faculty were recruited, including an Amercian gynaecologic oncologist as Head of Department, a South African professor to lead research training, and a Motswana OBGYN who had been sponsored by UB to train in South Africa, increasing total OBGYN specialist staff from 4 to 7. One of the existing OBGYN faculty members applied and was appointed Assistant Programme Director, responsible for the residency training programme. Pre-existing faculty had participated in the development of the curriculum and new faculty were oriented to the curriculum.

**Table 2:** Learning outcomes and assessment criteria

Learning Outcomes	Assesstment Criteria
1.0 Provides high quality patient care in OBGYN	1.1 Competently perform a clinical interview and physical examination relating to complex Obstetric and Gynaecologic problems
	1.2 Accurately identify and interpret clinical findings
	1.3 Succinctly synthesize clinical problems to formulate a working diagnosis
	1.4 Select and where needed perform relevant tests. Appropriately interpret and use results in patient care.
	1.5 Implement appropriate management plans based on best available evidence
	1.6 Refer patients for further specialised care as appropriate and co-manage patients in an interdisciplinary team to address complex clinical problems.
	1.7 Effectively educate and counsel patients and their families regarding identified clinical problems, their management and prognosis
	1.8 Plan appropriate follow up
	1.9 Maintain adequate clinical records of all practice events
	1.10 Perform appropriate, safe and precise OBGY surgeries
2.0 Medical Knowledge	2.1 Apply knowledge of biomedical, clinical and behavioural sciences in patient evaluation and management.
	2.2 Apply knowledge of advanced science of the reproductive system, its function and malfunction to patient management.
	2.3 Use analytical and investigative approaches in treatment planning using evidence based medicine
	2.4 Critically appraise literature: its quality relevance and utility in patient management
	2.5 Engage in continuing professional development activities
3.0 Interpersonal communication skills	3.1 Demonstrate good interpersonal communication skills with patients, their families and all members of the health care team; in both verbal and nonverbal format
4.0 Professionalism	4.1 Model compassionate care and professionalism
	4.2 Recognise the roles other health care workers play, treat them with respect and consult them appropriately.
	4.3 Provide leadership when called upon to do so
	4.4 Demonstrate accountability to the patient, society and the profession.
	4.5 Actively participate in teaching and upskilling of medical students, medical officers and other members of the healthcare team
5.0 Practice-based learning environment	5.1 Identify areas for personal improvement to enhance knowledge, attitude, skills
	5.2 Participate in quality improvement projects such as clinical audits and use evidence to improve patient care
	5.3 Produce an original research thesis
6.0 Rational use of resources	6.1 Practise cost-effective health care interventions without compromising patient care and clinical outcomes
	6.2 Advocate for resources for effective delivery of preventative and curative reproductive health services
	6.3 Promote preventative reproductive health services to avert costs related to morbidity

Twenty-six candidates interviewed in November 2019; given the high demand, the initial planned cohort of 3 residents was expanded to 4. All chosen candidates accepted admission and began training in January 2020. The MOHW provided salary support through existing mechanisms for all residents.

Our curriculum is tailored to specific disease profiles and OBGYN needs in Botswana, adjusted for the resource environment. A particular contextual need is increased training time in general surgery, because of limited back-up support. Similarly, because gynaecologic oncology subspecialty services are not widely available in the region, a high level of competence in gynaecologic oncology was deemed necessary. In view of the high human immunodeficiency prevalence, a robust understanding of cervical cancer screening, diagnosis, and staging, as well as advanced skills in vulvar cancer surgery and staging were deemed critical (Tables 3 and 4).

**Table 3:** Rotation allocation in the 4-year Obstetrics and Gynaecology Master in Medicine programme

Rotations	Duration (months)
General Obstetrics	15
General Gynaecology	12
General surgery	3
Intensive Care	1
Radiology	1
High-risk Obstetrics	4
Gynaecologic Oncology	4
Fertility Medicine & Gynaecologic Endocrinology	1
Urogynaecology	1
Research/Elective	2
Vacation	4

**Table 4:** Obstetrics and Gynaecology procedure minimums

Obstetrics		
Operative vaginal deliveries:		15
Caesarean sections:	200 of which the following subminima must be certified:	
	Repeat caesarean section	50
	Caesarean section for breech presentation	10
	Caesarean section for placenta praevia	5
	Caesarean section for a less than 1200 g baby	10
Ultrasound	150 of which the following subminima must be certified:	
Obstetrics	50	
	Abdominal gynaecology	50
	Transvaginal	50
**Gynaecology**		
Abdominal hysterectomy		50
Myomectomy		25
Adnexal laparotomy		25
Vaginal hysterectomy		5
Vaginal procedures (ie anterior repair; evacuations excluded)		10
Vulvar wide local excisions		15
Cone biopsy		5
Laparoscopic procedures	20 of which the following subminima must be certified:	
	Laparoscopic tubal ligation	5
	Diagnostic laparoscopy	5
	Operative adnexal laparoscopy	5
Long-acting contraceptive procedures		20

The UB gynaecologic oncologist bridged a substantial gap in gynaecologic cancer care and training. International academic collaborations were developed to bolster subspecialty training in gynaecologic oncology and address remaining gaps in urogynaecology, reproductive endocrinology, minimally invasive gynaecologic surgery, high-risk obstetrics, and ultrasound training. Complementing strong generalist training with regional and international subspecialty collaboration provides residents with a robust knowledge base and should provide adequate exposure to foster curiosity in subspecialty training.

As a new programme, we opted to assess trainee knowledge and clinical competence with annual examinations, which also provides early feedback to the programme on its quality. The OBGYN MMed research requirement was tailored such that residents produce a scientific manuscript of 2500 to 10,000 words as a thesis, with the intent to publish. Co-supervision of resident research by both junior and senior faculty, as well as external thesis review, was designed to improve overall departmental research capacity.

To date (approximately 8 months into the programme), the clinical rotations, OBGYN specific courses and requisite graduate medical education courses have been delivered as planned. The first round of resident continuous assessment using the milestone framework with input from all faculty members was completed six months into the academic year and feedback sessions with all residents were held.

An anonymous programme evaluation survey was distributed to all residents after the first semester and the results were reviewed with both residents and faculty so that timely improvements could be implemented. Such feedback included a desire for more immediate feedback during clinical rotations, clear role delineation in multi-level learner teams, and inclusion of an internal medicine rotation. Additionally, all residents have made appropriate progress on their research projects, having identified a proposal topic and embarking on their draft research proposals.

The local accreditation process was initiated with the Botswana Health Professions Council and the Botswana Qualifications Authority. External accreditation will be sought from the Colleges of Medicine of South Africa (CMSA) to provide international validation of programme quality. Internal and external UB academic programme review is scheduled every 5 years.

To promote retention in the public sector, all residents who receive government funding for their residency training were obliged to sign a commitment to a minimum of 4 years of public service. With MOHW as a key stakeholder in the development and financing of this programme, it is invested in retaining the graduates of the programme in public service. Additionally, the Southern Africa Development Community has an agreement in place preventing Batswana health professionals from being hired in another country, in an effort to prevent regional brain drain.

## Discussion

The initiation of the first OBGYN MMed residency training programme in Botswana fulfils a longstanding demand for in-country OBGYN training and supports efforts to increase human resource capacity in medicine in the country. Strong residency training programmes strengthen on-going medical training in multiple ways, including improving the medical student experience, improving clinical care, and developing research capacity. Sandwich programmes send senior residents abroad, just when they are best equipped to train medical students and junior residents, resulting in a void in the training hierarchy and senior-level clinical coverage. A fully in-country programme allows Batswana residents to contribute to education and health system strengthening in their own country.

A primary outcome of our programme is an increase in the number of OBGYN specialists in Botswana, who will be expected to play a critical role in health system improvement by increasing capacity for specialist care beyond tertiary centres [21–23]. A strong residency training programme rooted in evidence-based medicine will further facilitate the establishment of standard practice guidelines to enable the regulation of the quality of OBGYN services in the country. An in-country programme like ours confers the benefit of preparing OBGYNs to practice in their unique resource environment, while exposing them to cutting edge technologies to advance OBGYN capacity locally.

Similar in-country training programmes have demonstrated high retention of graduates in their public health and academic sectors [24, 25]. This may be attributed to acquisition of the necessary skills during training to effectively work in their environment. Additionally, residents may experience improved quality of life during in-country training compared to training abroad [6, 26]. A fully in-country training programme accords trainees support from their families and social networks, and hopefully reinforces their commitment to serve Botswana.

There are limitations in the generalizability of our experience. Other LMIC settings may require intense financial support if they do not have pre-existing allocated staff funding and a strong MOHW partner that supports resident salaries. We benefited from the institutional experience of launching successful MMed programmes and recruiting two senior faculty members with experience pioneering similar programmes in Sub-Saharan Africa. This addition to faculty in the department who had successfully pioneered undergraduate OBGYN medical training at UB was a substantial boost to capacity. Another limitation of our programme may be the ability to provide robust subspecialty training. At the time of planning the programme, international short trips were routine and UB had prior successful implementation of such collaborations. Changes in global travel patterns and restrictions may require development of alternative delivery of some subspecialty curricular content.

The success of our programme is yet to be proven, and the robustness of our curriculum and overall training programme is still to be assessed. Systematic evaluation of outcomes of our training programme is essential, including evaluation of the role of the programme in increasing access to reproductive health services, improving quality of care and ultimately reducing maternal morbidity and mortality. Short-term proxies of health system improvements include the number of OBGYN citizen specialists practicing in Botswana, waiting times for a consultation and surgery, and the proportion of districts with OBGYN specialists. A cost analysis of international versus in-country training, considering the staffing benefit during training and retention should be undertaken.

A concerted effort for retention of our graduates in public and academic sectors is critical to ensure the programme attains its aims of addressing the specialty healthworker shortage and leading efforts to improve the quality of care in the country. Beyond the mandatory 4 years of clinical service to the MOHW, retention in the public sector will require a competitive pay structure, and opportunities for a fulfilling career with a reasonable work-life balance. Creating opportunities for OBGYN subspecialty training may further contribute to improved job satisfaction and retention.

## Conclusion

Training OBGYNs in-country has many benefits to health systems in SSA and addresses significant human resource gaps. Curricula can be adjusted to local resource context yet achieve international standards through thoughtful design and purposeful collaborations. Evaluation of the impact of training programs on population health and health systems is essential.

## Data Availability

Not applicable, however, any additional information on programme proposal contents and accreditation process is available upon request.

## Abbreviations

CMSA: Colleges of Medicine of South Africa
LMICs: Low- and middle-income countries
MMed: Master of Medicine
MOHW: Ministry of Health and Wellness
OBGYN: Obstetrics and Gynaecology
OBGYNs: Obstetrician Gynaecologists
SSA: Sub-Saharan Africa
UB: University of Botswana
